# Methylglyoxal Impairs Insulin Secretion of Pancreatic *β*-Cells through Increased Production of ROS and Mitochondrial Dysfunction Mediated by Upregulation of UCP2 and MAPKs

**DOI:** 10.1155/2016/2029854

**Published:** 2015-12-07

**Authors:** Jinshuang Bo, Shiya Xie, Yi Guo, Chunli Zhang, Yanming Guan, Chunmei Li, Jianxin Lu, Qing H. Meng

**Affiliations:** ^1^Wenzhou Medical University School of Laboratory Medicine and Life Sciences, Wenzhou, Zhejiang 325035, China; ^2^Key Laboratory of Laboratory Medicine, Ministry of Education, Wenzhou, Zhejiang 325035, China; ^3^Zhejiang Provincial Key Laboratory of Medical Genetics, Wenzhou, Zhejiang 325035, China; ^4^Department of Laboratory Medicine, The University of Texas MD Anderson Cancer Center, Houston, TX 77030, USA

## Abstract

Methylglyoxal (MG) is a highly reactive glucose metabolic intermediate and a major precursor of advanced glycation end products. MG level is elevated in hyperglycemic disorders such as diabetes mellitus. Substantial evidence has shown that MG is involved in the pathogenesis of diabetes and diabetic complications. We investigated the impact of MG on insulin secretion by MIN6 and INS-1 cells and the potential mechanisms of this effect. Our study demonstrates that MG impaired insulin secretion by MIN6 or ISN-1 cells in a dose-dependent manner. It increased reactive oxygen species (ROS) production and apoptosis rate in MIN6 or ISN-1 cells and inhibited mitochondrial membrane potential (MMP) and ATP production. Furthermore, the expression of UCP2, JNK, and P38 as well as the phosphorylation JNK and P38 was increased by MG. These effects of MG were attenuated by MG scavenger N-acetyl cysteine. Collectively, these data indicate that MG impairs insulin secretion of pancreatic *β*-cells through increasing ROS production. High levels of ROS can damage *β*-cells directly via JNK/P38 upregulation and through activation of UCP2 resulting in reduced MMP and ATP production, leading to *β*-cell dysfunction and impairment of insulin production.

## 1. Introduction

Diabetes mellitus is a chronic and progressive metabolic condition characterized by hyperglycemia. According to statistics from International Diabetes Federation, as of 2013 an estimated 382 million people worldwide had diabetes, and an estimated 592 million are expected to have diabetes by 2035 [1, 2]. About 90% of the cases are type 2 diabetes, which features *β*-cell failure and chronic insulin resistance [3]. While type 1 diabetes is caused by loss of insulin secretion due to destruction of pancreatic *β*-cells [4], progressive *β*-cell failure, including disruption of *β*-cell function and reduction of *β*-cell mass, is the central component of the onset and progression of type 2 diabetes [5]. The mass reduction and dysfunction of *β*-cells have been shown in many studies to lead ultimately to insulin deficiency in patients with type 2 diabetes [3, 6]. Collectively, pancreatic *β*-cell dysfunction and reduced insulin secretion play an important role in the pathogenesis of both type 1 and type 2 diabetes.

Methylglyoxal (MG) is a highly reactive intermediate metabolite that normally is produced only in small amounts from basal carbohydrate, lipid, and protein metabolism [7, 8]. MG production is highly increased in hyperglycemic disorders such as diabetes mellitus [9–11]. It is believed to be a major precursor of advanced glycation end products that are involved in the pathogenesis of diabetes and diabetic complications [8]. Several studies have proved that MG can induce damage to tissues, including vascular endothelial and smooth muscle tissue and cells, in diabetes [12, 13]. However, data on the direct effects of MG on *β*-cells and insulin secretion are scarce.

Uncoupling protein (UCP) is a mitochondrial inner membrane protein that can decrease metabolic efficiency by dissipating the proton gradient in the mitochondrion from ATP synthesis [14]. UCP2, which was first described in 1997, is expressed in multiple tissues, including the pancreas [15]. Earlier studies demonstrated that UCP2 can decrease formation of reactive oxygen species (ROS) [16, 17] and regulate free fatty acid metabolism and transport [14, 18]. Increased UCP2 expression under oxidative stress may be a negative modulator of insulin secretion. Recent studies have shown that increased expression of UCP2 can suppress glucose-stimulated insulin secretion [19].

Oxidative stress, which may induce *β*-cell apoptosis and decrease *β*-cell mass, is involved in the pathological process of diabetes [20, 21]. Pancreatic *β*-cells are susceptible to ROS, and the action of ROS on these cells is considered a potential mechanism of glucose toxicity in diabetes [20]. ROS can activate the c-Jun N-terminal kinase (JNK) and the P38 mitogen-activated protein kinase (MAPK), both of which may induce mitochondrial dysfunction and then cell apoptosis [22, 23]. We and others have shown that increased MG production increases ROS production [24, 25].

Although the pathological effects of MG in diabetes and diabetic complications are known, there is little evidence showing whether MG has an impact on insulin secretion by *β*-cells. In the present study, we investigated whether MG impairs insulin secretion by *β*-cells and the potential mechanisms involved. Furthermore, we examined whether N-acetyl cysteine (NAC), an MG scavenger, could prevent the deleterious effects of MG.

## 2. Materials and Methods

### 2.1. Cell Culture and Reagents

MIN6, a mouse insulinoma cell line, purchased from Shanghai Bioleaf Biotech Company Limited (Shanghai, China), was cultured in Dulbecco modified essential medium (Gibco, Carlsbad, CA) supplemented with 10% fetal bovine serum (Gibco, Carlsbad, CA), 2 mM L-glutamine, 100 U/mL penicillin, 100 *μ*g/mL streptomycin, and 50 *μ*M *β*-mercaptoethanol at 37°C in 5% CO_2_. INS-1, a rat insulinoma cell line, was obtained from American type culture collection (ATCC, USA). The cells were grown in RPMI-1640 medium (Gibco, Carlsbad, CA) supplemented with 10% fetal bovine serum, 10 mM 4-(2-hydroxyethyl)-1-piperazineethanesulfonic acid (HEPES), 2 mM L-glutamine, 50 *μ*M *β*-mercaptoethanol, 1 mM sodium pyruvate, 100 U/mL penicillin, and 100 *μ*g/mL streptomycin at 37°C in atmosphere containing 95% air and 5% CO_2_. MIN6 cells were used throughout of the whole experiments to demonstrate our hypothesis while INS-1 cells were used in some of the experiments to confirm the major findings. MG, NAC, and hydrogen peroxide (H_2_O_2_) were purchased from Sigma (St. Louis, MO). The following regents were used: Mouse/Rat Insulin ELISA Kit (Shanghai Westang Biotech, Shanghai, China); Reactive Oxygen Species Assay Kit, Mitochondrial Membrane Potential Assay Kit with JC1, and caspase-3 Activity Assay Kit (all three, Beyotime Institute of Biotechnology, Haimen, China); Cell Counting Kit-8 (CCK-8; Dojindo Laboratories, Kumamoto, Japan); Annexin V-FITC Apoptosis Detection Kit (Nanjing KeyGEN Biotech, Nanjing, China); ATP Determination Kit (Invitrogen, Grand Island, NY); UCP2 antibody (Beijing Biosynthesis Biotechnology, Beijing, China); p-JNK antibody, JNK antibody, p-P38 antibody, and P38 antibody (all four, Cell Signaling Technology, Beverly, MA); and *β*-actin antibody and goat anti-rabbit IgG (both, Bioworld Technology, St. Louis Park, MN).

### 2.2. Quantitation of Insulin Secretion

MIN6 or INS-1 cells were plated in Corning 24-well tissue culture plates at 2 × 10^5^ per well and allowed to attach overnight. The medium was then replaced with the same medium containing various concentrations of MG (0.05 mM or 0.1 mM) with or without NAC (0.6 mM) and incubated for 3 h. The group that cells were incubated without MG and NAC was defined as control group. The cells were washed with Krebs Ringer Buffer (KRB; 5 mM NaHCO_3_, 129 mM NaCl, 1 mM MgCl_2_, 4.8 mM KCl, 1.2 mM KH_2_PO_3_, 2.5 mM CaCl_2_, 10 mM HEPES, and 0.2% bovine serum albumin; pH 7.4) and then incubated with KRB for 30 min. The medium was then replaced with KRB containing 5 mM glucose and the cells incubated for another 2 h. The supernatants were collected for determination of insulin secretion. The cells then were maintained with KRB containing 25 mM glucose for 2 h, and again the supernatants were collected for measurement of insulin secretion. The insulin concentration in the supernatants was measured by using the Mouse/Rat Insulin ELISA Kit according to the manufacturer's instructions. The results were normalized to the protein concentrations detected by the Bicinchoninic Acid (BCA) Protein Assay Kit (Beyotime Institute of Biotechnology).

### 2.3. Detection of Cell Apoptosis

MIN6 cells were plated in 24-well plates and incubated with medium containing various concentrations of MG (0.05 mM or 0.1 mM) or H_2_O_2_ (0.2 mM) with or without NAC (0.6 mM) for 3 h. The cells were then collected and washed twice with PBS. The cell suspensions were incubated with Annexin V with fluorescein isothiocyanate (FITC) and propidium iodide (PI) from the Annexin V-FITC Apoptosis Detection Kit for 15 min. Apoptosis was detected by a FACSCalibur Flow Cytometer (BD Biosciences, San Jose, CA). Cells in the lower right quadrant with FITC positivity but PI negativity were at early stage of apoptosis. The rate of cells in the lower right quadrant was considered as apoptosis rate.

Caspase-3 activity was evaluated with the caspase-3 Activity Assay Kit according to the manufacturer's protocol. In brief, MIN6 cells were subjected to lysis with the ice-cold buffer provided in this kit and the supernatants were incubated with caspase-3 substrate AC-DEVD-*ρ*NA on a 96-well plate. The activity of caspase-3 was determined by an Automatic Microplate Reader at 405 nm and the results were normalized to the protein concentrations detected by the Bradford Protein Assay Kit (Beyotime Institute of Biotechnology).

### 2.4. Determination of ROS Production and Mitochondrial Membrane Potential

MIN6 or INS-1 cells were seeded in Corning 24-well tissue culture plates at 2 × 10^5^ per well and allowed to attach overnight. Medium containing various concentrations of MG (0.05 mM or 0.1 mM) or H_2_O_2_ (0.2 mM) with or without NAC (0.6 mM) was applied and the cells were incubated for 3 h. After that, cells were treated with ROS Detection Solution (1 : 1000 dilution; Reactive Oxygen Species Assay Kit) for 20 min at 37°C and then washed according to the manufacturer's protocol. The ROS levels were determined by the fluorescence intensity of dichlorodihydrofluorescein diacetate (DCF) with a FACSCalibur Flow Cytometer and fluorescence microscope (Nikon, Tokyo, Japan).

Mitochondrial membrane potential (MMP) was assayed by using JC1 (from the Mitochondrial Membrane Potential Assay Kit with JC1) according to the manufacturer's protocol. The fluorescence was determined with a FACSCalibur Flow Cytometer. Cells in the bottom right gate have low MMP, and the rate of these cells that is low MMP rate can reflect the level of decreased MMP.

### 2.5. Measurement of ATP Production

MIN6 cells were plated in Corning 6-well tissue culture plates at 1 × 10^6^ per well and allowed to attach overnight. The medium was then replaced with medium containing various concentrations of MG (0.05 mM or 0.1 mM) with or without NAC (0.6 mM) and incubated for 3 h. Cells were collected and washed twice with PBS. The sediment was resuspended with ATP extracting solution (100 mM Tris, 4 mM EDTA, pH adjusted to 7.75 with glacial acetic acid) after centrifugation (500 ×g, 4 min). This cell resuspension was heated at 100°C for 90 s and subjected to centrifugation (10000 ×g, 1 min). The supernatants were extracted for detection of ATP production with the ATP Determination Kit according to the manufacturer's protocol; ATP activity was determined by an Automatic Microplate Reader.

### 2.6. Determination of* Ucp2* mRNA

Total RNA from MIN6 cells treated with MG (0.05 mM or 0.1 mM) with or without NAC (0.6 mM) was extracted by using Trizol (Invitrogen) and reverse-transcribed into cDNA with the PrimeScript RT reagent Kit (Takara Biotechnology, Dalian, China) according to the manufacturer's instructions. Real-time quantitative polymerase chain reaction (PCR) was performed with the QuantiTect SYBR Green PCR Kit (Qiagen, Hilden, Germany). Reactions were performed with the 7500 Standard program on a 7500 Fast Real-Time PCR System (Applied Biosystems, Grand Island, NY). Cycling parameters were as follows: 95°C 5 min followed by 40 cycles of 95°C 10 s + 60°C 30 s. The relative expression of* Ucp2* mRNA was normalized by using the 2^−ΔΔCt^-method relative to *β*-actin. The specific primers were as follows: forward 5′-GTCGGAGATACCAGAGCACT-3′, reverse 5′-GTGACCTGCGCTGTGGTACT-3′. The primers for *β*-actin were forward 5′-GAGACCTTCAACACCCCAGC-3′, reverse 5′-CCACAGGATTCCATACCCAA-3′. All primers were synthesized by Sangon Biotech (Shanghai, China).

### 2.7. Determination of UCP2, p-JNK, JNK, p-P38, and P38 Protein Expression

After incubation with various concentrations of MG (0.05 mM or 0.1 mM) or H_2_O_2_ (0.2 mM) with or without NAC (0.6 mM) for 1, 2, and 3 hours, MIN6 cells were washed with PBS and subjected to lysis in radioimmunoprecipitation assay (RIPA) lysis buffer (Beyotime Institute of Biotechnology). Protein concentration was determined by the BCA Protein Assay kit. Proteins were heated at 100°C for 8 min, separated with sodium dodecyl sulfate on a 10% polyacrylamide gel by electrophoresis (SDS-PAGE), and transferred to a nitrocellulose membrane (0.45 *μ*m, Beyotime Institute of Biotechnology). The membrane blots were blocked with 5% (w/v) nonfat dried milk for 2 h and incubated at 4°C overnight with the following primary antibodies: anti-UCP2 antibody (1 : 400), anti-p-JNK antibody, anti-JNK antibody (1 : 1000), anti-p-P38 antibody (1 : 1000), anti-P38 antibody (1 : 1000), and anti-*β*-actin antibody (1 : 1000). The membranes were incubated with goat anti-rabbit IgG (1 : 5000) for 1 h at room temperature. After washing for 1 h, the membranes were visualized with electrochemiluminescence (ECL) reagent by the Western blotting detection system (Bio-Rad, Hercules, CA).

### 2.8. Statistical Analyses

Data were analyzed by analysis of variance (ANOVA) and two-tailed Student's *t*-test and results are expressed as mean ± standard deviation. All experiments were repeated independently at least three times. Statistical analysis was performed by SPSS 17.0 software (Chicago, IL). A *p* value <0.05 was considered statistically significant.

## 3. Results

### 3.1. MG Reduced Insulin Secretion by MIN6/INS-1 Cells

**Figure 1:**
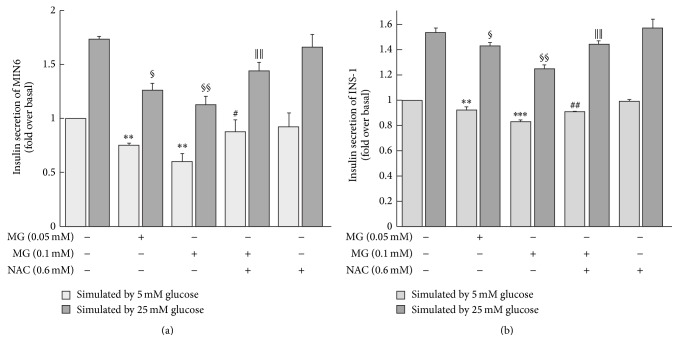
Effects of MG on insulin secretion by MIN6 (a) and INS-1 (b) cells. Insulin secretion stimulated by low glucose concentration (5 mM) or high glucose concentration (25 mM) was reduced by preincubation with MG. The effects of MG on MIN6/INS-1 were dependent on the concentration of MG and were reversed by coincubation with NAC. ^*∗*^ *p* < 0.05, ^*∗∗*^ *p* < 0.01, and ^*∗∗∗*^ *p* < 0.001 compared to control group (0 mM MG + 0 mM NAC) with 5 mM glucose stimulated. ^#^ *p* < 0.05, ^##^ *p* < 0.01 compared to the higher MG group (0.1 mM MG + 0 mM NAC) with 5 mM glucose stimulated. ^§^ *p* < 0.05, ^§§^ *p* < 0.01 compared to control group (0 mM MG + 0 mM NAC) with 25 mM glucose stimulated. *p* < 0.01 compared to the higher MG group (0.1 mM MG + 0 mM NAC) with 25 mM glucose stimulated.

Incubation of MIN6 cells with 0.05 or 0.1 mM MG for 3 h significantly reduced the cells' insulin secretion under conditions of low glucose (5 mM) stimulation (0.75 ± 0.02 or 0.60 ± 0.07, resp., versus 1.00 ± 0.00 fold change over baseline, *p* < 0.01; Figure 1(a)). The suppressive effect of MG on insulin secretion was dose dependent. Coincubation of MG with NAC reversed the impairment in glucose-stimulated insulin secretion induced by MG (0.87 ± 0.12 versus 0.60 ± 0.07, *p* < 0.05; Figure 1(a)). Likewise, MG (0.05 mM or 0.1 mM) decreased insulin secretion under conditions of high glucose (25 mM) stimulation compared with the control group (1.26  ±  0.06 or 1.12 ± 0.08, resp., versus 1.73 ± 0.03 fold change over baseline, *p* < 0.05 and *p* < 0.01, resp.; Figure 1(a)). This inhibitory effect of MG on insulin secretion by MIN6 was attenuated by coincubation with NAC (1.44 ± 0.08 versus 1.12 ± 0.08, *p* < 0.01; Figure 1(a)). However, the effects of MG on insulin secretion stimulated by 5 mM glucose or 25 mM glucose were not significantly different. Similarly, insulin secretion of INS-1 under 5 mM or 25 mM glucose stimulation was reduced after incubation with MG (0.05 mM or 0.1 mM) for 3 h. The inhibitory effects of MG were reversed by NAC in INS-1 cells (Figure 1(b)).

### 3.2. MG Increased Apoptosis of MIN6 Cells

**Figure 2:**
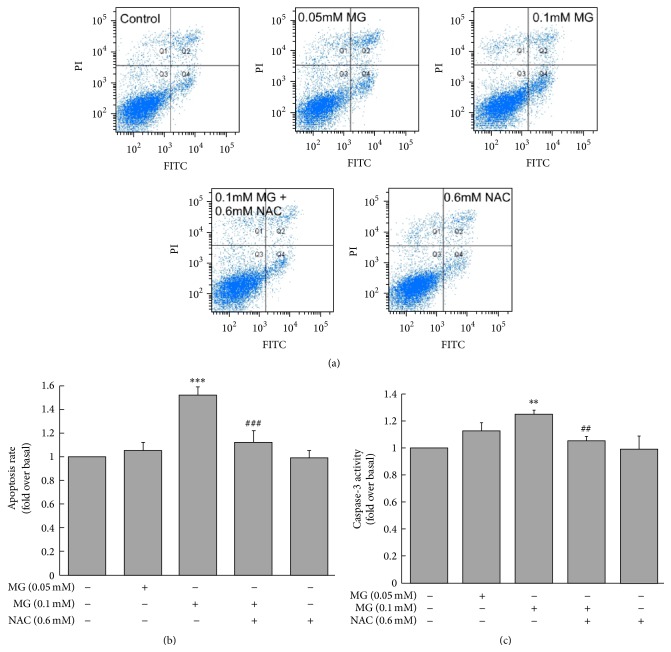
Effects of MG on MIN6 cell apoptosis. Apoptotic rate was higher in MG-treated MIN6 than in controls, and this effect was relieved by coculturing with NAC. (a) Apoptosis was measured by Annexin V-FITC/PI assay, and FITC-positive but PI-negative cells were quantified by flow cytometer. (b) Statistical results of (a). (c) Caspase-3 activity. ^*∗∗*^ *p* < 0.01, ^*∗∗∗*^ *p* < 0.001 compared to control group (0 mM MG + 0 mM NAC). ^##^ *p* < 0.01, ^###^ *p* < 0.001 compared to the higher MG group (0.1 mM MG + 0 mM NAC).

Treatment of MIN6 cells with 0.1 mM MG for 3 h increased cell apoptosis rate compared with the control group (1.52 ± 0.07 versus 1.00  ±  0.00 fold change over baseline, *p* < 0.001; Figures 2(a) and 2(b)). This effect was prevented by coincubation with NAC (1.12 ± 0.10 versus 1.52 ± 0.07, *p* < 0.001; Figures 2(a) and 2(b)). Similarly, incubation of MIN6 cells with 0.1 mM MG for 3 h markedly increased caspase-3 activity compared with the control group (1.24 ± 0.04 versus 1.00 ± 0.00 fold change over baseline, *p* < 0.01; Figure 2(c)). Again, the increase of caspase-3 activity was attenuated by coincubation with NAC (1.05 ± 0.04 versus 1.24 ± 0.04, *p* < 0.01; Figure 2(c)).

### 3.3. MG Increased ROS Production and Reduced MMP in *β*-Cells

**Figure 3:**
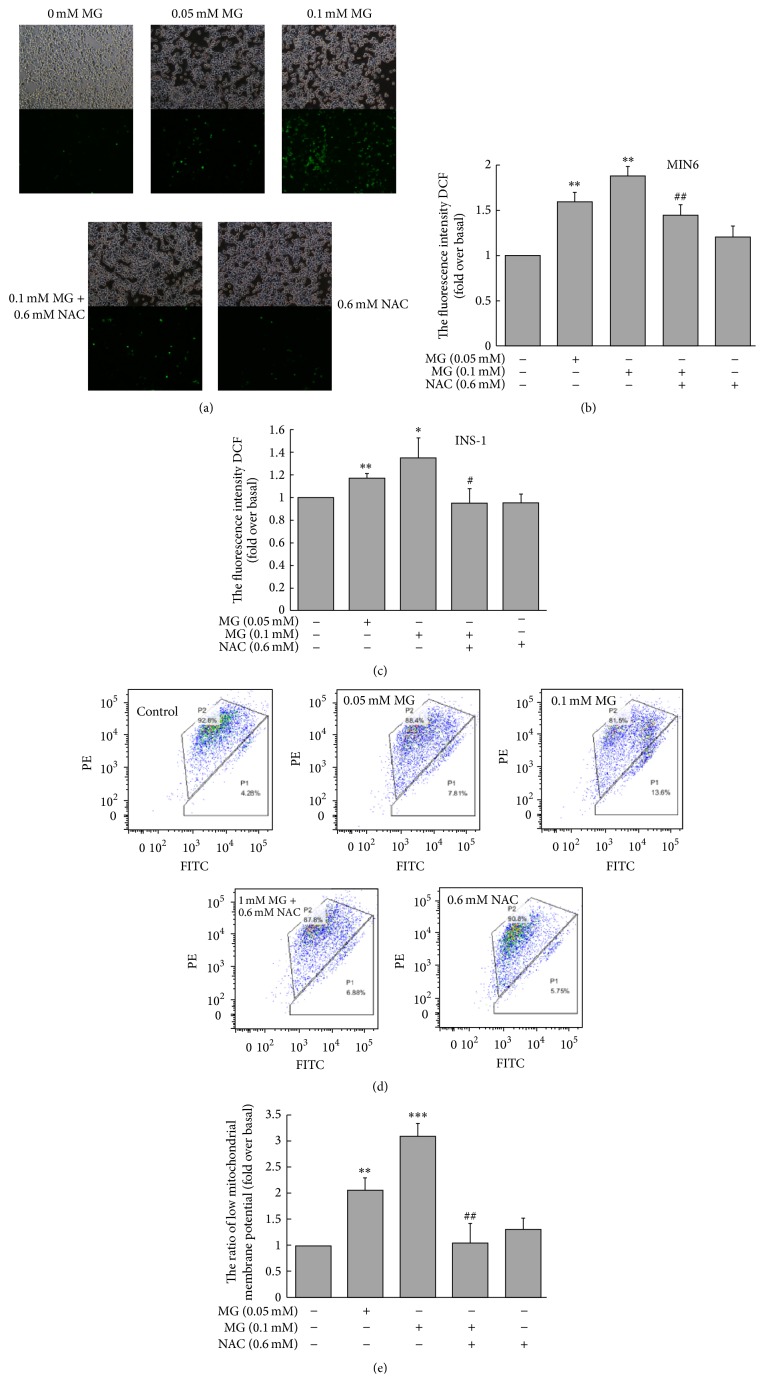
Effects of MG on the production of ROS in MIN6/INS-1 cells and MMP in MIN6 cells. ((a), (b), and (c)) ROS production was significantly increased by MG, as detected by fluorescence microscope ((a) for MIN6; 100x) and flow cytometer ((b) for MIN6; (c) for INS-1). ((d) and (e) for MIN6) Incubation with MG for 3 h increased the rate of low MMP (the bottom right gate). This illustrated that MG significantly decreased MMP. The effects of MG on MIN6 were reversed by coincubation with NAC. ^*∗*^ *p* < 0.05, ^*∗∗*^ *p* < 0.01, and ^*∗∗∗*^ *p* < 0.001 compared to control group (0 mM MG + 0 mM NAC). ^#^ *p* < 0.05, ^##^ *p* < 0.01 compared to the higher MG group (0.1 mM MG + 0 mM NAC).

Incubation of cultured MIN6 cells with MG (0.05 mM or 0.1 mM) for 3 h significantly increased the fluorescence intensity of DCF, an indicator of ROS production, as shown by fluorescence microscopy and flow cytometry (Figures 3(a) and 3(b)). The increased fluorescence intensity of DCF was attenuated by coincubation with NAC (Figures 3(a) and 3(b)). Similarly, ROS production of INS-1 was increased after incubation with MG (0.05 mM or 0.1 mM) for 3 h, which was reversed by coincubation with NAC (Figure 3(c)). The rate of low MMP was increased by exposure to 0.05 or 0.1 mM MG for 3 h, indicating that MG decreased MMP in MIN6 cells; MMP was restored by coculturing with NAC (Figures 3(d) and 3(e)).

### 3.4. MG Reduced ATP Production in MIN6 Cells

**Figure 4:**
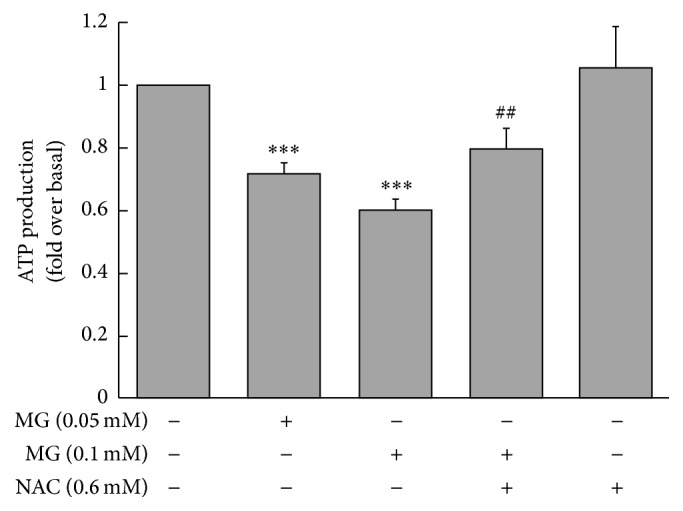
Effects of MG on the production of ATP by MIN6 cells. Reduction of ATP production by MG depended on MG concentration. The effects of MG were reversed by coincubation with NAC. ^*∗∗∗*^ *p* < 0.001 compared to control group (0 mM MG + 0 mM NAC). ^##^ *p* < 0.01 compared to the higher MG group (0.1 mM MG + 0 mM NAC).

The production of ATP was markedly reduced in MIN6 cells incubated with 0.05 or 0.1 mM MG for 3 h compared with the control group (0.72 ± 0.03 or 0.60 ± 0.04, resp., versus 1.00 ± 0.00 fold change over baseline, *p* < 0.001; Figure 4). The inhibitory effect of MG on ATP production was attenuated by coincubation with NAC (0.80 ± 0.06 versus 0.60 ± 0.04, *p* < 0.01; Figure 4).

### 3.5. MG Increased* UCP2* mRNA and Protein Expression in MIN6 Cells

**Figure 5:**
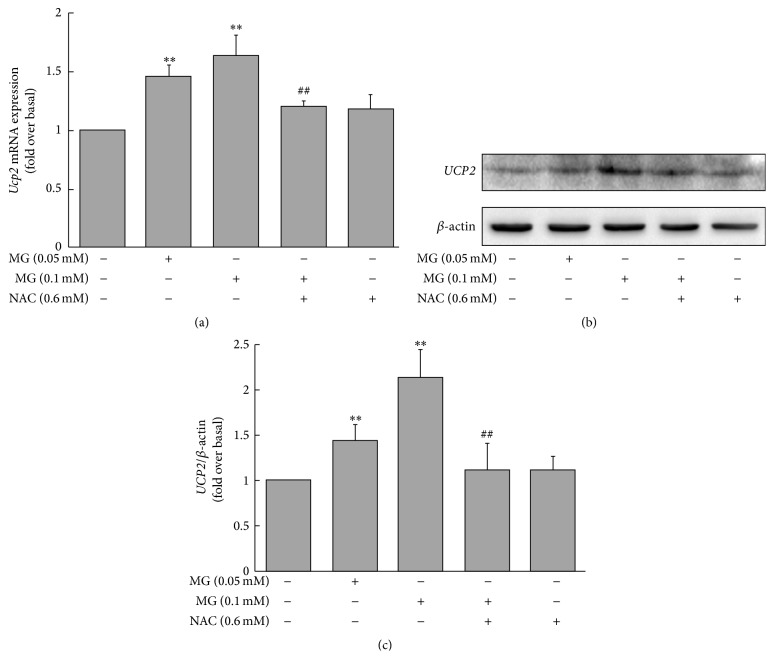
Effects of MG on* Ucp2* mRNA and UCP2 protein levels in MIN6 cells. The expression of* Ucp2* mRNA (a) and UCP2 protein ((b) and (c)) was increased by treatment with MG. Cells were incubated with 0.05 or 0.1 mM MG for 3 h. Results are fold change compared to baseline (0 mM MG + 0 mM NAC). The effects of MG were reversed by coincubation with NAC. ^*∗∗*^ *p* < 0.01 compared to control group (0 mM MG + 0 mM NAC). ^#^ *p* < 0.05, ^##^ *p* < 0.01 compared to the higher MG group (0.1 mM MG + 0 mM NAC).

The* Ucp2* mRNA level was markedly increased in MIN6 cells incubated with 0.05 or 0.1 mM MG for 3 h compared with the control group (1.46 ± 0.12 or 1.63 ± 0.18, resp., versus 1.00 ± 0.00 fold change over baseline, *p* < 0.01; Figure 5(a)). The effects of MG on* Ucp2* mRNA expression were attenuated by coincubation of NAC (1.20 ± 0.06 versus 1.63 ± 0.18, *p* < 0.01; Figure 5(a)). Similar effects were observed in UCP2 protein levels between the group of cells treated with 0.05 or 0.1 mM MG and the controls (1.44 ± 0.18 or 2.13 ± 0.31, resp., versus 1.00 ± 0.00 fold change over baseline, *p* < 0.01; Figures 5(b) and 5(c)). Coincubation of cultured cells with NAC restrained the increase of UCP2 protein levels induced by MG (1.21 ± 0.28 versus 2.13 ± 0.31, *p* < 0.05; Figures 5(b) and 5(c)).

### 3.6. MG Increased Expression of p-JNK, JNK, p-P38, and P-38 in MIN6 Cells

**Figure 6:**
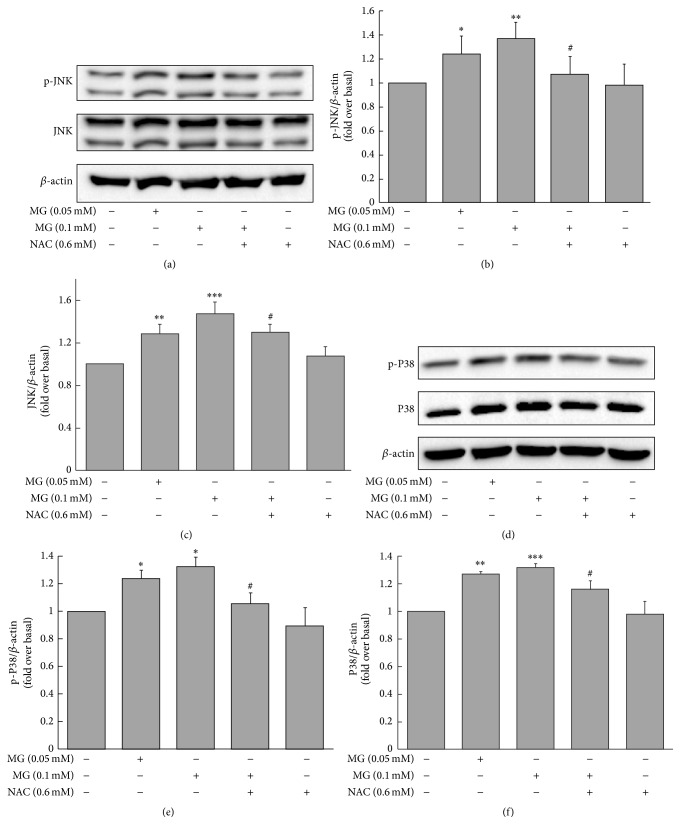
Effects of MG on expression of p-JNK, JNK, p-P38, and P38 proteins in MIN6 cells. The expression of p-JNK and JNK (a) and p-P38 and P38 (d) was increased by MG. Cells were incubated with 0.05 or 0.1 mM MG for 3 h. Results shown in (b), (c), (e), and (f) are fold change compared to baseline (0 mM MG + 0 mM NAC). The effects of MG on MIN6 were reversed by coincubation with NAC. ^*∗*^ *p* < 0.05, ^*∗∗*^ *p* < 0.01, and ^*∗∗∗*^ *p* < 0.001 compared to control group (0 mM MG + 0 mM NAC). ^#^ *p* < 0.05 compared to the higher MG group (0.1 mM MG + 0 mM NAC).

**Figure 7:**
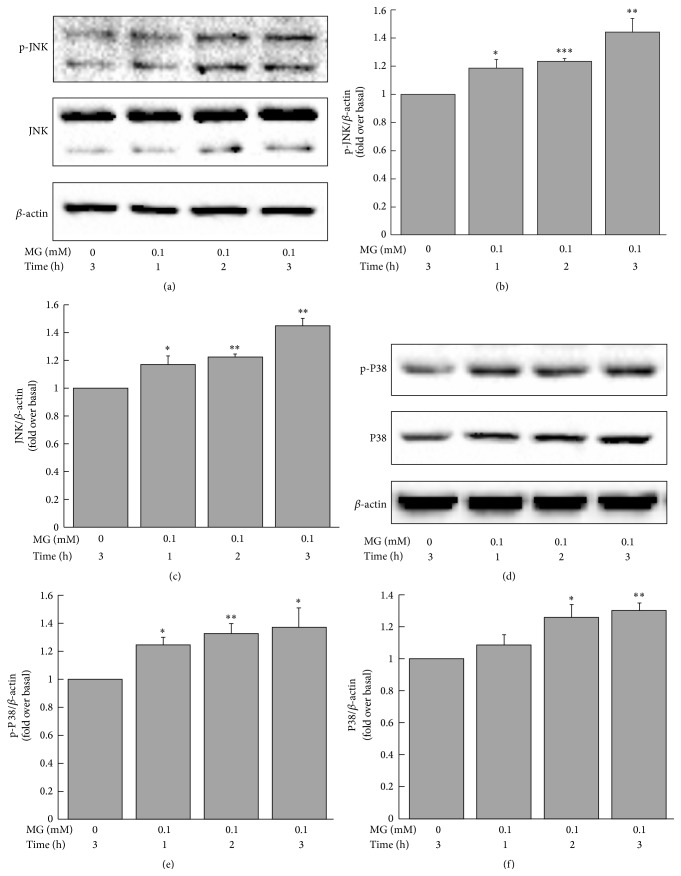
Time-dependent effects of MG on expression of p-JNK, JNK, p-P38, and P38 proteins in MIN6 cells. The expression of p-JNK and JNK (a) and p-P38 and P38 (d) was increased after treatment with MG for 1 h. Cells were incubated with 0.1 mM MG for 1, 2, and 3 h. Results shown in (b), (c), (e), and (f) are fold change compared to baseline (0 mM MG). ^*∗*^ *p* < 0.05, ^*∗∗*^ *p* < 0.01, and ^*∗∗∗*^ *p* < 0.001 compared to control group (0 mM MG).

Treatment of MIN6 cells with MG (0.05 or 0.1 mM) for 3 h significantly stimulated the expression of p-JNK protein (1.25 ± 0.15 or 1.34 ± 0.13, resp., versus 1.00 ± 0.00 fold change over baseline, *p* < 0.05 and *p* < 0.01, resp.) and JNK protein (1.29 ± 0.09 or 1.48 ± 0.11, resp., versus 1.00 ± 0.00 fold change over baseline, *p* < 0.01 and *p* < 0.001, resp.) compared to the control group (Figures 6(a)–6(c)). The effects of MG on p-JNK or JNK were attenuated by coincubation with NAC (for p-JNK: 1.08 ± 0.15 versus 1.34 ± 0.13, *p* < 0.05; for JNK: 1.30 ± 0.08 versus 1.48 ± 0.11, *p* < 0.05; Figures 6(a)–6(c)). Similarly, there were parallel differences in p-P38 protein and P38 protein expression between cells treated with 0.05 or 0.1 mM MG and controls (for p-P38: 1.24  ±  0.06 or 1.33 ± 0.07, resp., versus 1.00 ± 0.00 fold change over baseline, *p* < 0.05; for P38: 1.28 ± 0.01 or 1.32 ± 0.03, resp., versus 1.00 ± 0.00 fold change over baseline, *p* < 0.01 and *p* < 0.001, resp.; Figures 6(d)–6(f)). These effects were diminished by coculturing with NAC (for p-P38: 1.06 ± 0.08 versus 1.33 ± 0.07, *p* < 0.05; for P38: 1.16 ± 0.06 versus 1.32 ± 0.03, *p* < 0.05; Figures 6(d)–6(f)). Indeed, MAPK (p-JNK, JNK, p-P38, and P38) upregulation induced by MG started after treatment with 0.1 mM MG for 1 h and continued to increase as the time prolonged (Figures 7(a)–7(e)).

### 3.7. H_2_O_2_ Increased ROS Production, Apoptotic Rate, and Expression of MAPK in MIN6 Cells

**Figure 8:**
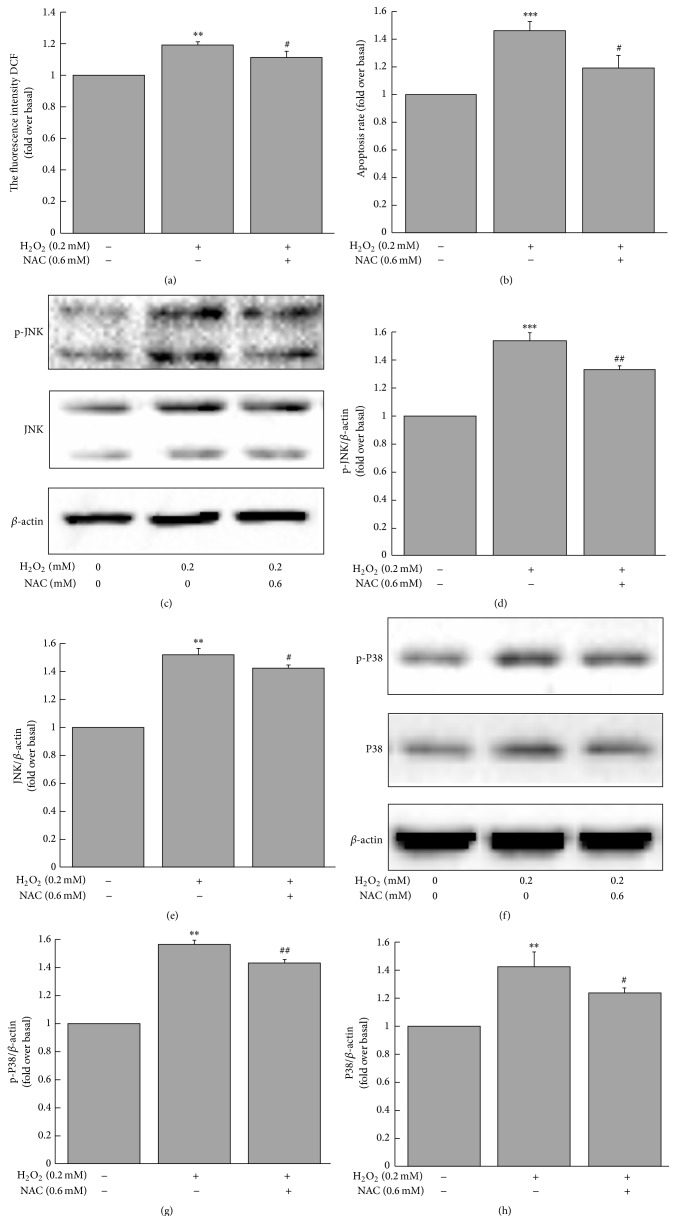
Effects of H_2_O_2_ on ROS production (a), apoptotic rate (b), and expression of MAPK in MIN6 cells. Results shown in (a), (b), (d), (e), (g), and (h) are fold change compared to baseline (0 mM H_2_O_2_ + 0 mM NAC). ^*∗∗*^ *p* < 0.01, ^*∗∗∗*^ *p* < 0.001 compared to control group (0 mM H_2_O_2_ + 0 mM NAC). ^#^ *p* < 0.05, ^##^ *p* < 0.01 compared to H_2_O_2_ group (0.2 mM H_2_O_2_ + 0 mM NAC).

Incubation of MIN6 cells with 0.2 mM H_2_O_2_ for 3 h significantly increased cellular ROS production (Figure 8(a)), apoptotic rate (Figure 8(b)), and MAPK protein expression (Figures 8(c)–8(h)). These effects were attenuated by coincubation with 0.6 mM NAC (Figures 8(a)–8(h)). 0.6 mM NAC may be not sufficient to offset the effects of 0.2 mM H_2_O_2_. The effects of NAC may be more distinct if increasing the NAC concentration.

## 4. Discussion

Despite the recognized pathological role of MG in diabetes and diabetic complications, little previous study has, to our knowledge, investigated the possible impact of MG on insulin secretion or the mechanism underlying any such effect. In this study, we provide evidence that MG impaired insulin secretion in cultured MIN6 and INS-1 cells. The effects of MG on MIN6 and INS-1 cells are dose dependent and can be prevented by NAC, an antioxidant and MG scavenger [12, 26].

After demonstrating that MG suppressed insulin secretion by MIN6 and INS-1 cells, we investigated the potential mechanisms underlying this effect. We found that MG increases ROS production and apoptosis in these cells and that this increase of ROS production and apoptosis was attenuated by NAC. These findings suggest that MG may aggravate oxidative stress and apoptosis in pancreatic *β*-cells. Previous studies have demonstrated that MG can induce ROS production and lead to oxidative stress in various cells [25, 27, 28]. Oxidative stress can induce apoptosis and increase chemical modification of proteins and lead to tissue damage. Oxidative stress and resultant tissue damage are hallmarks of chronic disease and cell death, and diabetes is no exception [29]. Furthermore, excessive ROS levels can damage *β*-cells and induce *β*-cell dysfunction [30]. Thus, increased ROS production and apoptosis by MG are responsible for the MG-induced impairment of *β*-cells and insulin secretion.

Mitochondrial dysfunction is a central contributor to *β*-cell failure. UCP2 is a member of a family of proteins that are located in the mitochondrial inner membrane and act as proton channels to uncouple mitochondrial oxidative phosphorylation [31]. UCP2 is involved in various physiological and pathological processes including insulin secretion [14]. Increased ROS can induce UCP2 upregulation [14]. UCP2 facilitates proton leak to reduce the MMP and thus attenuates ATP synthesis. Reduced ATP production results in the dysfunction of *β*-cells [31]. It has been reported that UCP2 negatively regulates ATP production and insulin secretion [14]. Indeed, optimal ATP synthesis is necessary for efficient activation of the triggering pathway of insulin secretion [32]. We have shown that MG increased UCP2 expression and reduced MMP and ATP production. Our findings indicate that ROS activate UCP2, which results in proton leak across the mitochondrial inner membrane and reduced *β*-cell ATP production, leading to impairment of insulin secretion.

JNK and P38 MAPKs are members of the complex superfamily of MAP serine/threonine protein kinases, which are activated by phosphorylation. JNK and P38 MAPKs are stress-activated kinases and are responsive to stress-inducing stimuli such as ROS [30, 33]. It has been reported that oxidative stress induces damage to *β*-cells by activating stress-sensing pathways via JNK and P38 [23, 34]. When isolated rat islets were exposed to oxidative stress, the JNK, P38, and protein kinase C pathways were activated. Activation of the JNK pathway can induce *β*-cell apoptosis and decrease pancreatic and duodenal homeobox factor-1 (PDX-1) activity and subsequent suppression of insulin gene transcription and resultant inhibition of insulin secretion in the diabetic state [34]. In the present study, we demonstrate that not only MG increased apoptosis in MIN6 and INS-1 cells but it also increased the expression and activation of JNK and P38. The effects of MG on MAPK upregulation were earlier than the biological effects and continued to increase as the time was prolonged. We speculate, therefore, that increasing the expression of JNK/P38 is another mechanism by which MG can cause cell damage and impair insulin secretion. Since H_2_O_2_ can induce ROS production, MIN6 cells were incubated with H_2_O_2_ to verify the effects of ROS in MIN6 cells. Results showed that apoptotic rate and MAPK protein expression were increased. These further confirmed that the effects of MG in pancreatic *β*-cells might be related to ROS production. Studies have shown that the effects of MG can be attenuated by NAC [12, 35]. Our findings are consistent with the previous studies. Our findings may explain the previous observation that chronic infusion of MG reduces insulin secretion and the development of type 2 diabetes in SD rats and expand the underlying mechanisms of MG-induced impaired insulin secretion [36, 37].

**Figure 9:**
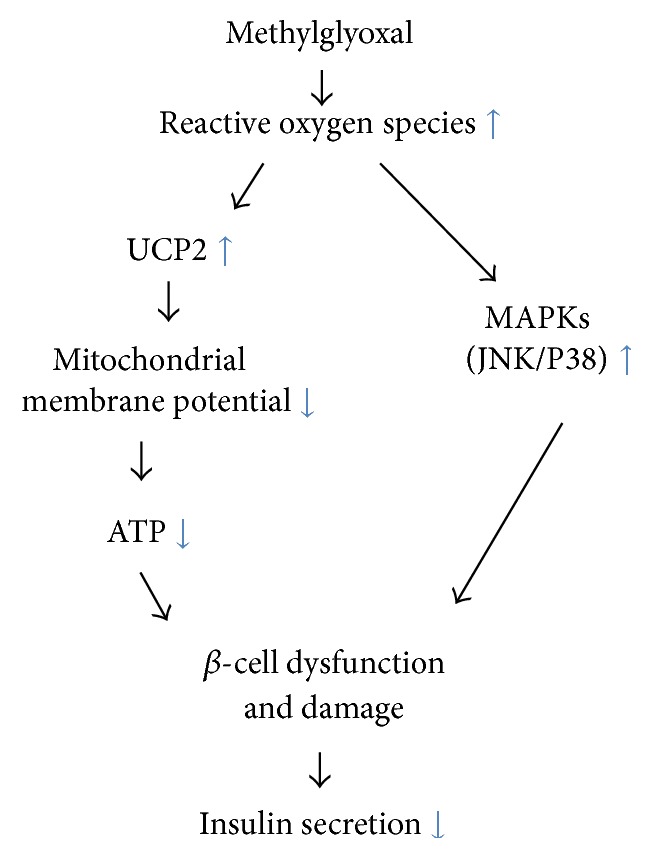
Schematic representation of proposed signaling pathways suggested by the results of this study. Methylglyoxal increases production of reactive oxygen species (ROS), which then reduces the mitochondrial membrane potential (MMP) and ATP production via UCP2 upregulation. UCP2 upregulation in turn leads to *β*-cell damage and, ultimately, impairment of insulin secretion. ROS also may induce *β*-cell damage and impair insulin secretion directly via upregulation of MAPKs (JNK/P38).

In conclusion, the data presented here demonstrate that MG can damage insulin secretion of pancreatic *β*-cells through increased production of ROS and apoptosis, mitochondrial dysfunction, and upregulation of UCP2 and MAPKs. Based on our findings, we postulate that MG induces increased production of ROS, which may trigger the upregulation of both UCP2 and JNK/P38. Increased JNK and P38 can directly damage *β*-cells causing cell dysfunction. UCP2 can reduce MMP and ATP synthesis, resulting in *β*-cell damage and impairment of insulin secretion (Figure 9). These findings reveal a novel pathological role and potential mechanisms of MG in the pathogenesis of diabetes. These findings also provide evidence that MG could be a new therapeutic target in diabetes management and that improving *β*-cell function should be given attention in the treatment of diabetes.
